# A Framework for the Comparative Assessment of Neuronal Spike Sorting Algorithms towards More Accurate Off-Line and On-Line Microelectrode Arrays Data Analysis

**DOI:** 10.1155/2016/8416237

**Published:** 2016-04-27

**Authors:** Giulia Regalia, Stefania Coelli, Emilia Biffi, Giancarlo Ferrigno, Alessandra Pedrocchi

**Affiliations:** Neuroengineering and Medical Robotics Laboratory, Department of Electronics, Information and Bioengineering, Politecnico di Milano, 20133 Milano, Italy

## Abstract

Neuronal spike sorting algorithms are designed to retrieve neuronal network activity on a single-cell level from extracellular multiunit recordings with Microelectrode Arrays (MEAs). In typical analysis of MEA data, one spike sorting algorithm is applied indiscriminately to all electrode signals. However, this approach neglects the dependency of algorithms' performances on the neuronal signals properties at each channel, which require data-centric methods. Moreover, sorting is commonly performed off-line, which is time and memory consuming and prevents researchers from having an immediate glance at ongoing experiments. The aim of this work is to provide a versatile framework to support the evaluation and comparison of different spike classification algorithms suitable for both off-line and on-line analysis. We incorporated different spike sorting “building blocks” into a Matlab-based software, including 4 feature extraction methods, 3 feature clustering methods, and 1 template matching classifier. The framework was validated by applying different algorithms on simulated and real signals from neuronal cultures coupled to MEAs. Moreover, the system has been proven effective in running on-line analysis on a standard desktop computer, after the selection of the most suitable sorting methods. This work provides a useful and versatile instrument for a supported comparison of different options for spike sorting towards more accurate off-line and on-line MEA data analysis.

## 1. Introduction

Simultaneous multisite recordings using Microelectrode Arrays (MEAs) coupled to cultured neuronal networks are a widely applied approach in the field of* in vitro* electrophysiology [1–3]. This technology overcomes the drawbacks of single-cell recording techniques, by sampling the electrical activity of a neuronal culture from multiple sites in a noninvasive way. This allows long-term studies of extracellular neuronal action potentials (i.e., spikes) and high frequency sequences of spikes (i.e., bursts) recorded at each electrode. Moreover, neuronal dynamics at the network level, such as array-wide bursts barrages (i.e., network bursts), can be observed thanks to the multiple sites of recording [1]. Given that typical electrode diameters are comparable to cells size or bigger (i.e., 20–30 *μ*m), each electrode is able to sense the extracellular activity of multiple cells simultaneously. As a consequence, specific signal processing methods (i.e., spike sorting algorithms) are needed to identify the network activity at a single-cell level, before proceeding with the analysis of spike trains (e.g., burst and network burst detection). Spike sorting algorithms are designed to address this task, based on the assumption that the coupling between an individual cell and its respective electrode creates a unique spike shape [4, 5]. Applications where the focus is on the spike timing analysis [6] or that require discrimination between the activities of neurons of different origin [7–9] are examples of studies that particularly benefit from spike sorting.

Many algorithms with different levels of complexity and automaticity have been proposed to sort neuronal spikes [5]. Most of the methods start with a feature extraction step, where prominent spike waveform features are computed. After an optional dimensionality reduction, a clustering step is applied on the extracted spike features to identify groups of spikes belonging to the same cell [5, 10]. Other methods rely on the temporal matching or on the correlation of spike waveforms with spike templates, without the feature extraction phase [11, 12].

Despite many efforts to tackle the spike sorting problem, it is still difficult to identify the best algorithm with large generality and also to define which spike sorter is the most appropriate under specific circumstances [13]. As reported earlier, the experimental protocol, the acquisition setup, the culture type, and the unique coupling of cells and electrodes within the same culture shape the electrode data in a peculiar way, making it inevitable to use data-centric methods for the classification of neuronal spikes [10].  Table 1 reports the heterogeneity of some of the spike sorting algorithms found in the literature and in custom or commercial software [14–34]. Except for a few instances [31], these tools do not incorporate alternative methods for spike sorting process (i.e., feature extraction and clustering) but usually apply one method indiscriminately to all the electrodes [20, 33]. This prevents an immediate comparison of different algorithms on the same data.

Neuronal spikes recorded with MEAs can be sorted with two different approaches: (i) off-line, which means that spikes are sorted after the acquisition and storage of raw voltage traces, or (ii) on-line, which means that spikes are sorted during data acquisition. In the first case, the information about the spikes collected throughout the recording is available to the algorithm; in the other case, only information available up to the current point in time can be exploited to sort a spike. Although in off-line modality spike waveforms are classified with a better accuracy [4], an extensive amount of time, together with a massive data transmission rate and storage space, is often required for this task [11]. An immediate data analysis is crucial not only in closed-loop situations (on-line real-time processing) [35] but also for open-loop long-lasting and/or high-throughput experiments, taking into account that standard MEAs can generate tens of gigabytes of raw data per day [24, 36]. Moreover, this approach facilitates for experimental scientists having a glance at the results of their ongoing experiment. On-line spike classification methods have been incorporated into few commercial (e.g., McRack software by Multi Channel Systems GmbH, Spike2 by Cambridge Electronic Design Ltd.) or custom acquisition software [30, 32], as shown in Table 1. However, as stated for the vast majority of the off-line toolboxes, the available on-line implementations do not allow the user to select the appropriate algorithms to apply from a pool of methods. This aspect may be detrimental for sorting accuracy, due to the poor generality of spike sorting methods.

To our knowledge it is difficult to find a spike sorting framework that incorporates alternative methods for all the spike sorting processing steps and that allows on-line analysis with any selected method. To this aim, in this work different spike sorting methods proposed in the literature and suitable for on-line analysis have been selected and integrated within a software environment familiar to the MEA users, that is, Matlab. The tool is to provide the users with the possibility to select a method according to the data set at hand, optionally different for each electrode of the same MEA. Here, the working principle of each algorithm is described together with metrics and the evaluation flow used to assess the performance of each method. To validate the tool, accuracy performances of the implemented methods on neuronal signals (both simulated and real recordings) are reported and discussed. Finally, the work reports a comparison of runtimes of the implemented spike sorting algorithms during MEA data acquisition with a test-bed setup, showing that on-line operations are feasible.

## 2. Materials and Methods

### 2.1. Algorithms

**Figure 1:**
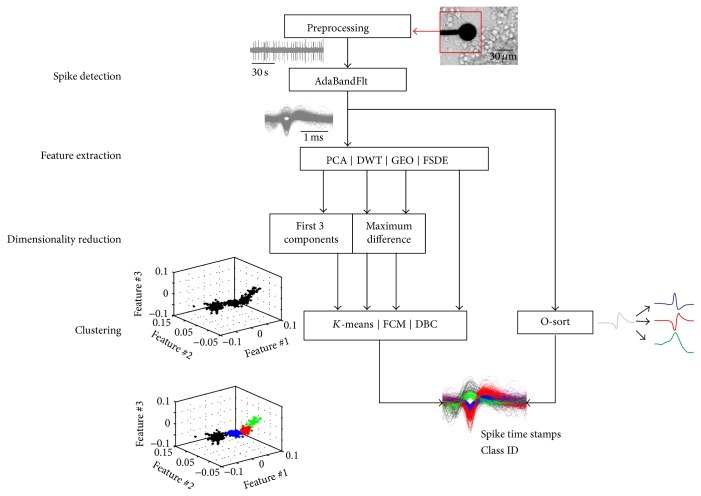
Scheme of the spike sorting processing algorithms incorporated in this work. For each electrode the raw signal is preprocessed before the subsequent spike detection by a threshold-based algorithm (i.e., AdaBandFlt [24]). The feature extraction can be performed with four different methods (i.e., principal component analysis (PCA), Discrete Wavelet Transform (DWT), geometric features (GEO), and First and Second Derivative Extrema (FSDE)) followed by a dimensionality reduction step that retains the relevant features. Three clustering algorithms are implemented to automatically cluster spike features (i.e., *K*-means, fuzzy-*C*-means (FCM), and density-based clustering (DBC)). As an alternative, a template matching algorithm (O-sort) groups the spikes as soon as they are detected.

An overview of the processing blocks and implemented methods is presented in Figure 1.

#### 2.1.1. Spike Detection and Alignment

To attain accurate spike sorting, spike waveforms have to be properly detected and aligned. The most common methods to detect spikes apply a threshold to the voltage of the input signal, computed as a multiple of the standard deviation of the signal over a predefined window. In this work, spike waveforms data provided as inputs to spike classifiers were obtained by means of an adaptive threshold-based algorithm, that is, “AdaBandFlt,” fully described in [24]. This method updates the current noise level esteem every 1 second and detects spikes by comparison with both a positive and negative threshold (set as ±4 times the noise level), with appropriate checks to avoid detecting twice a single event [24]. When a sample crosses the threshold, a 3 ms window (1 ms before the threshold crossing and 2 ms after it) is applied to the signal and the result is saved as a spike. Spikes are then aligned to the point of the maximum amplitude [5, 20, 37].

#### 2.1.2. Algorithms for Building Blocks of Spike Sorting Process

Four feature extraction algorithms, coupled to suitable dimensionality reduction methods and to three feature clustering methods, and one waveform clustering method (i.e., without a feature extraction phase) have been selected for implementation (Figure 1).

*(1) Feature Extraction (FE)*. The aim of FE step is to extract representative features from a set of events to judge the differences between spike waveforms. The following clustering is facilitated if spikes are projected into compact and distant groups in the feature space [5].  Table 2 reports details about the four FE methods included in this work, pertaining their analysis domain, their occurrence in spike sorting publications, and the need of an off-line training phase before the on-line application of the methods.

*(a) Principal Component Analysis (PCA)*. Principal component analysis is the benchmark feature extraction method in spike sorting of neural signals [38]. To obtain spike features, eigenvalue decomposition of the covariance matrix of the data (i.e., spike waveforms) is computed. Then, eigenvectors associated with the directions onto which projected data display the largest variance are used as the “principal components” (PCs). Each spike is represented by a series of PC coefficients *c*_*i*_:

(1) $$\begin{array}{c} c_{i}=\overset{N}{\underset{n=1}{\sum }}{PC}_{i}\left(\;n\;\right)*s\left(\;n\;\right), \end{array}$$

where *s*(*n*) is the waveform of the spike, PC_*i*_ is the *i*th PC, and *N* is the number of samples contained in a spike waveform. The dimensionality is usually reduced by only keeping the first few PCs, thus decreasing the computation time of PC coefficients calculation [5]. The computation of PCs matrix is usually performed using the entire set of acquired spikes. However, the method can be applied on-line by computing the PCs on a first set of acquired spikes (training set) and then performing a dot product between the PCs matrix and each new incoming waveform [5, 30].

*(b) First and Second Derivative Extrema (FSDE)*. A feature extraction method based on the first and second derivative calculation of the incoming spike profile was recently proposed as an efficient and low computational complexity solution [33]. The derivatives are computed as the difference between successive sample points, according to (2) for the first derivative (FD) and (3) for the second derivative (SD). Consider

(2) FDn=sn−sn−1,

(3) SDn=FDn−FDn−1,

where *s*(*n*) is the waveform of the spike. The positive peak of the first derivative (FD_max_) and the negative and the positive peaks of the second derivative (SD_min_ and SD_max_) are then extracted as spike features. This method does not require a training phase or a dimensionality reduction step, which makes it very suitable for on-line operation.

*(c) Geometric Features (GEO)*. Geometric features relate to the shapes of the spike waveforms and usually require low complexity operations [4]. In this work, seven geometric features have been considered, including (i) the positive amplitude, (ii) the negative amplitude, (iii) the peak-to-peak amplitude, (iv) the positive area, (v) the negative area, (vi) the ratio between the positive and negative area, and (vii) the maximum slope. After feature computation, a dimensionality reduction step determines which subset of features best represents the differences among waveforms. As for PCA, on-line GEO feature extraction can be computed after a training step on a first set of spikes.

*(d) Discrete Wavelet Transform (DWT)*. The DWT is a multiresolution technique which provides good time resolution at high frequencies and good frequency resolution at low frequencies. It has been shown to have the potential of outperforming PCA when spikes differ mainly in small details which are not captured by the few first components [39]. As in PCA, performing the DWT on a spike waveform provides a set of coefficients which can then be reduced and clustered to achieve spike classification. The coefficients obtained are the result of subsequent high- and low-pass filtering of the spike waveform at *j* different scales (i.e., filter bandwidths). The spike waveforms can then be described with a vector *v*:

(4) $$\begin{array}{c} v=\left\{\;a_{-j},d_{-j},d_{-\left(j-1\right)},\ldots ,d_{-1}\;\right\}, \end{array}$$

where *a*_−*j*_ is the output of the low-pass filter (approximation coefficients) at the *j*th scale and *d*_−*j*_, *d*_−(*j*−1)_,…, *d*_−1_ are the outputs of the high-pass filter at each scale (detail coefficients) [40]. In this work, Haar wavelet has been chosen as the default wavelet option, thanks to its computational efficiency and similarity to biphasic spike shapes [5, 20]. Sincethe DWT yields the same number of coefficients as samples in the original spike, a dimensionality reduction step is needed. As for PCA and GEO, the DWT-based FE can be applied on-line: the best subset of coefficients is chosen on a training data set and then these coefficients are retrieved from the DWT of incoming new spikes.

*(2) Dimensionality Reduction*. Dimensionality reduction is a critical step in spike sorting. Indeed, adding dimensions improves the accuracy of the subsequent clustering only up to a certain point, after which adding more dimensions can cause the performance of the clusterer to degrade [5]. One reason for this may be that dimensions along which the data points are not well separated introduce noise or confusion into the clusterer. In this work, after PCA, the first three PCs are retained to reduce dimensionality, which were known to capture most of the spike waveform variance [13]. To select the most suitable combination of both GEO and DWT features we implemented the “maximum difference” method proposed by Gibson and colleagues [13]. This method provided good accuracy and low complexity, thus reducing the time needed for data reduction. Briefly, given a vector containing features from an incoming spike, this algorithm computes the element by element difference between this vector and the vector related to the previous spike and memorizes the indices corresponding to the three largest difference values. The coefficients which presented the highest variability over a training set of spikes are identified as the coefficients that will be given as input to the clustering phase.

*(3) Spike Clustering*. An ideal clustering algorithm for on-line analysis should be (i) automatic (i.e., no need to set the number of clusters* a priori*), (ii) nonparametric (i.e., the user does not have to set arbitrary threshold values for algorithm parameters), and (iii) able to classify the incoming spikes without* a priori* knowledge. In this work, we selected for implementation two feature clustering methods (fuzzy-*C*-means and density-based clustering) and one template matching method (O-sort) fulfilling most of these requirements and the *K*-means, a benchmark off-line feature clustering method.  Table 3 summarizes the features of the selected methods. The following section details the implementation of each method in the proposed framework.

*(a) K-Means*. *K*-means clustering assigns a data point to a cluster according to the minimum Euclidean distance between the point and the centroid of the cluster. The main benefit of using this method in spike sorting is that it is a very simple and fast algorithm [5], yet it is supervised, since it requires inputting the number of clusters, usually unknown in advance. Different approaches have been proposed to automatically determine the number of clusters [41, 42]. Here we chose to maximize a structural index, that is, the “PBM index”:

(5) $$\begin{array}{c} PBM\left(\;k\;\right)={\left(\;\frac{1}{K}*\frac{E_{1}}{E_{k}}*S_{k}\;\right)}^{2}, \end{array}$$

where *K* is the number of clusters; *S*_*k*_ is the maximum separation (i.e., distance) between cluster centers; *E*_1_ is the sum of distances between all the points and the cluster center when *k* = 1; *E*_*k*_ is the sum of distances between the feature points and the *k*th cluster center [42]. In principle, it would be possible to adapt the algorithm for on-line operation, including a training period which defines the cluster centroids on a set of spikes and determines the number of clusters, followed by an on-line classification, which computes the distance of each new spike from the centroids. However, this would only be appropriate for stationary data because of its “hard” clustering [5].

*(b) Fuzzy-C-Means (FCM)*. In fuzzy clustering, every spike belongs to all possible classes, with different degrees of membership. The higher the membership of a spike to a given class is, the more likely the spike belongs to that class. Thus, the classification is performed according to the membership values of a spike to each cluster, which depends on the Euclidean distance from the cluster centroid and on the degree of fuzziness [43]. In the defuzzification phase, a spike is labeled according to the maximum membership value, provided that it overcomes a minimum threshold, otherwise the spike is not classified [16]. Unlike the *K*-means clustering, the “fuzziness” of the classification makes the method more suitable for on-line clustering, because it accounts for the varying nature of the data and does not classify outliers (noise) thanks to defuzzification. After a training period in which the centers of the cluster are identified, it is possible to perform the classification of the incoming spike by defuzzification [30]. To automatically define the number of clusters (*C*) during the training, the classification can be performed with different values of *C* and then, for each cluster configuration, the fuzzy clustering validity (*S*) is computed and minimized with respect to *C* [16]. This index is expressed as the ratio between the compactness and the separation of the clusters and can be explicitly written as in

(6) $$\begin{array}{c} S=\frac{\sum_{i=1}^{c}\sum_{j=1}^{N}\mu_{ij}^{m}{\left\|c_{i}-s_{j}\right\|}^{2}}{N*\min_{\mathrm{ik}}{\left\|c_{i}-c_{j}\right\|}^{2}}, \end{array}$$

where *μ*_*ij*_^*m*^ is the membership value of spike *j* belonging to cluster *i*, *m* is the degree of fuzziness (*m* > 1), *N* is the total number of spikes, and *c*_*i*_ and *s*_*j*_ are the center of cluster *i* and the position of spike *j*, respectively. In this work, the membership threshold for defuzzification was empirically set to 1/*C* + 0.1 (e.g., if *C* = 2 and the maximum membership value of a spike is lower than 0.6, the spike is not classified).

*(c) Density-Based Clustering (DBC)*. This feature clustering method was proposed by Cheng and colleagues [26, 27] as a classification method suitable for on-line operation and not requiring the setting of any threshold value (i.e., nonparametric). Moreover, in contrast to FCM and *K*-means algorithms, which provide round shaped clusters due to the Euclidean metric, no assumption on the shape of the cluster is made by DBC. The algorithm starts with a training phase in which a density distribution is built from the feature space. To this aim, each dimension of the feature space is firstly quantized into *N* different levels; then the feature space is divided into *N* ^*d*^ cells (*d* = dimensionality of the features). When a spike is detected, the density value of the cell containing the projected spike and of its surrounding cells increases according to a discrete spatial kernel. After computing the density distribution of the training spikes, a label is given to the cells corresponding to a local maximum of the density and to their surrounding cells. As the result, the entire density map is divided in an unsupervised way into several clusters corresponding to the local peaks of the density. Then, a further step can be introduced to merge the initial clusters [26, 27]. In the proposed implementation, two clusters are merged if the distance between their peaks and the peak density values are below a threshold. During on-line classification, an incoming spike is classified immediately by the look-up-table, provided that it falls within the mapped volume of the feature space (otherwise it is not classified).

*(d) O-Sort*. O-sort is a template matching algorithm presented by Rutishauser and colleagues [11]. The method is unsupervised, automatic, and designed to run in on-line mode, without the need of a training phase. The distance between an incoming spike and the already stored mean waveforms is computed. Then, the minimum of this distance indicates the class of the spike, provided that this is lower than a value (*T*_*M*_), otherwise a new cluster is introduced. If the distance between any cluster pair is lower than a value (*T*_*S*_), the clusters are merged into a single one. The authors use *T*_*M*_ = *T*_*S*_ = *N*〈*σ*〉^2^ to compute the thresholds, where *N* is the number of samples for each spike and 〈*σ*〉^2^ is the variance of the data computed over a sliding window (~1 min). Moreover, they suggest to multiply *T* by a correction factor, *c*, to take into account systematic changes of spike shapes [11]. Different from FCM and DBC, which may not include a new spike in any class, O-sort assigns a class to every input spike. To evaluate the method we discarded clusters with a number of spikes lower than a threshold (i.e., 5 spikes in 1-minute long signal), in order to account for the presence of small clusters of noise.

### 2.2. Test Data

To validate the implemented code, the methods have been applied to simulated signals and real MEA recordings.

**Figure 2:**
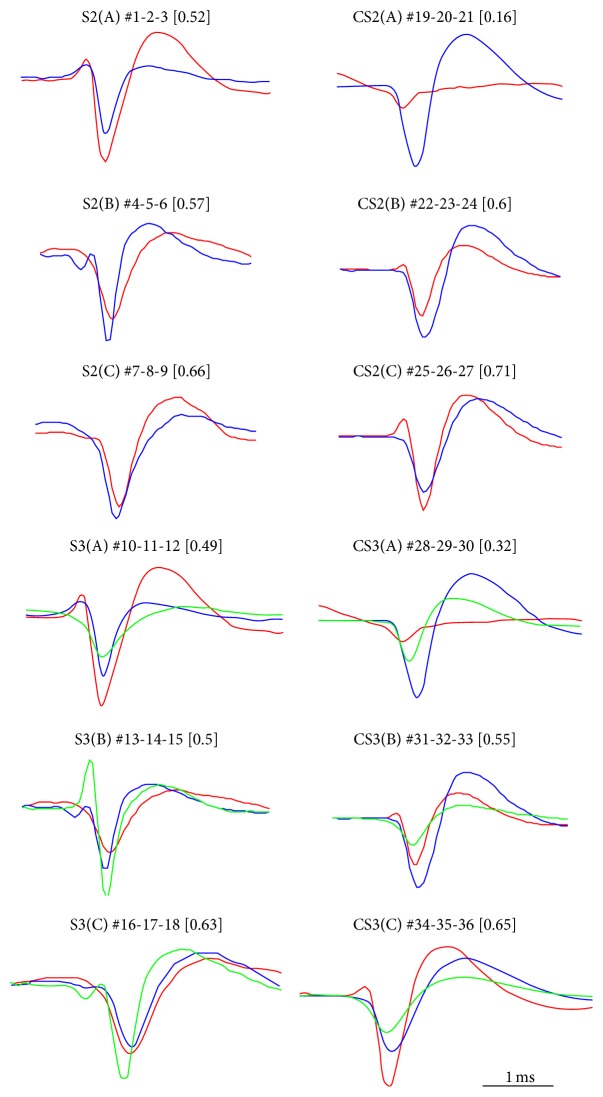
Features of the simulated data set. Spike waveforms were selected from a database of averaged spike waveforms obtained from spontaneous activity recorded in hippocampal and cortical* in vitro* neuronal networks by MEA. For each group of waveforms depicted in the figure, signals with three different SNR (4, 3, and 2) were simulated, obtaining a total of 36 signals. Each set of waveforms is associated with an ID name (e.g., S2(A)), the ordinal position in the data set (e.g., #1-2-3), and the mean Bray-Curtis similarity (BCS) between the waveforms (e.g., [0.52]).

*(1) Simulated Data*. Simulated raw data traces were generated by using average spike waveforms obtained from spontaneous activity recorded at 25 kHz from hippocampal and cortical dissociated cultures in our laboratory (see [44] for a reference about culturing and recording protocol), as done in [13, 20, 31]. In order to have a “ground truth” against which algorithm outcomes were compared, each waveform had been previously labeled according to the classification performed by experienced researchers. To simulate background noise, MEA signals without spikes were randomly selected from a set of 42 electrodes raw data. Noise was band-pass-filtered (2nd order Butterworth filter) between 200 Hz and 3000 Hz, and then it was normalized and overlapped to the simulated waveforms. Each signal contains waveforms belonging to either 2 or 3 different groups and a number of spikes between ~600 and 1000 over 60 seconds (i.e., average firing rate of single units between 3 Hz and 8 Hz, to mimic real recordings [44]). The occurrence of spikes were determined randomly from a uniform distribution, setting a refractory period of 2 ms. Three different levels of signal-to-noise ratio (SNR) were simulated (i.e., 2, 3, and 4 [20, 24]) rescaling the noise on the simulated signal to mimic the realistic levels of* in vitro* MEA recordings. SNR was defined as the ratio between the mean amplitude of the spike waveforms in the signal and the mean of the peak-to-peak amplitude of noise calculated over a 1-second window. A total of 36 data sets were thus obtained, whose features are reported in Figure 2, together with the Bray-Curtis similarity index, a measure of the similarity among the waveforms in each data set (see (7)). Consider

(7) $$\begin{array}{c} BCS\left(\;x,y\;\right)=1-\frac{\sum_{i}^{}\left|x\left(i\right)-y\left(i\right)\right|}{\sum_{i}^{}\left|x\left(i\right)\right|-\left|y\left(i\right)\right|}, \end{array}$$

where *x* and *y* are the two spike waveforms being compared and *N* is the number of sample points. BCS is computed between all the possible neuron pairs and then averaged. BCS lies in the range (0-1), with 1 corresponding to identical signals [33].

*(2) Real Data*. To test the algorithm performances in a realistic scenario, where the “ground truth” is unavailable, data sets of real MEA recordings were used. Extracellular recordings from hippocampal and cortical cultures grown on 60-channel MEAs (Ti200/30iR, Multi Channel Systems GmbH) had been previously carried out with a 60-channel MEA acquisition system (MEA1060 and USB-ME64, Multi Channel Systems GmbH) [44]. Recordings were sampled at 25 kHz and filtered (200 Hz–3 kHz, 2nd order Butterworth) before spike detection. The data set used in this work is composed of waveforms from 10 signals 120 seconds long.

### 2.3. Implementation of the Framework

The algorithms were implemented and evaluated in Matlab (version R2008b, The Mathworks). Scripts are in Matlab native language apart from part codes written in C language and running in Matlab as MEX-files. Source code for MEX-files was written using Microsoft® Visual C++ 2008 Express Edition. Graphical user interfaces (GUI) were designed using the graphical user interface development environment (GUIDE) of Matlab. To convert the file format generated by the acquisition software of our commercial acquisition platform (*∗*.mcd) to Matlab format (*∗*.mat), the “Neuroshare” API library of functions has been employed (freely downloadable at http://neuroshare.sourceforge.net/), as done in other MEA analysis frameworks [45].

### 2.4. Performance Evaluation

**Figure 3:**
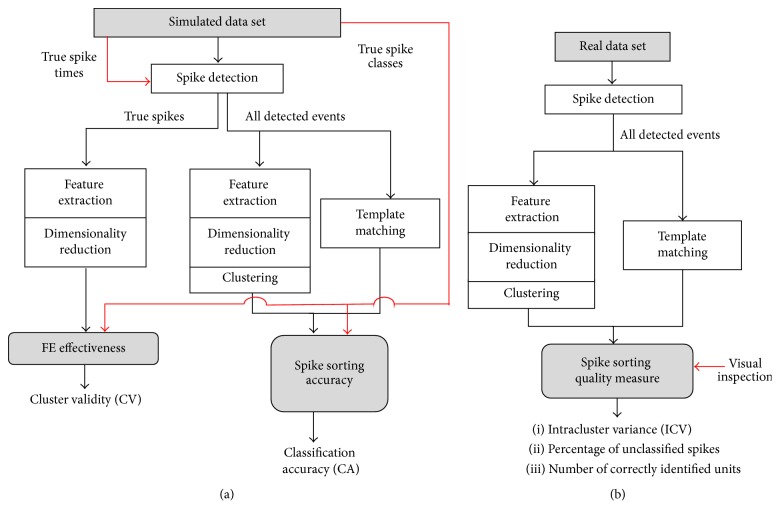
Performance assessment flow. (a) Scheme of the performance assessment procedure employed to evaluate the simulated data set in presence of “ground truth,” obtaining the cluster validity index and the classification accuracy. (b) Scheme of the performance assessment procedure employed to evaluate the set of real signals without a “ground truth,” obtaining the intracluster variance and parameters judged by visual inspection.

The evaluation scheme adopted to assess the performance of the algorithms on simulated and real signals is depicted in Figure 3. For methods requiring a training phase, the training was performed on the first detected spikes (~1/3 of the total number of spikes) for each signal, and then spikes were classified on the fly.

#### 2.4.1. Indexes of Performance

To test the effectiveness of spike sorting methods on simulated data sets, two indexes were employed:

(1) Cluster validity (CV), which is the ratio of the between-cluster to the within-cluster distance. This index was calculated as in (8) using the “ground truth” labels of the data points:

(8) $$\begin{array}{c} CV=\frac{\min_{i=1\cdots K,\;\;j=i+1\cdots K}{\left\|{CC}_{i}-{CC}_{j}\right\|}^{2}}{\left(1/N\right){\sum_{i=1}^{K}\sum_{A\in N_{i}}\left\|A-{CC}_{i}\right\|}^{2}}, \end{array}$$

where CC_*i*_ is the center of cluster of spikes produced by neuron *N*_*i*_, *N* is the total number of spikes, *K* is the number of neurons simulated in the recording, and *A* is the feature vector. The higher the cluster validity, the better the classes separation, which eases the task of the following clustering step [42].

(2) The rate of classification accuracy (CA) is defined as the percentage of the number of correctly classified spikes over the total number of input spikes [29], as in

(9) $$\begin{array}{c} CA\%=\frac{\text{number of correctly classifies events}}{\text{total number of spikes}}*100 \end{array}$$

The accuracy of the methods was evaluated without discarding the false positives of the detection (total number of spikes = true positives + false positives). In this way, we tried to mimic the real condition, where unavoidably a small percentage of the detected spikes represents noise. Thus, CA could reach 100% only if all true spikes are correctly clustered and if no false positives exist or all the false positives are left unclassified by the sorter.

To compare algorithm performances on real signals, two measures were used:

(1) Intracluster variance (ICV), given by the following [12]:

(10) $$\begin{array}{c} ICV=\frac{1}{N_{i}}\overset{N_{i}}{\underset{j=1}{\sum }}{\left(\;v_{j}-\mu_{i}\;\right)}^{2}, \end{array}$$

where *v*_*j*_ is the *j*th spike in the *i*th cluster, *μ*_*i*_ is its mean template, and *N*_*i*_ is the number of spike in the cluster *i*. Thus, the lower the value of ICV (meaning that the cluster is compact), the lower the probability of misclassification in each cluster.

(2) Comparison with expert's visual inspection of the results [46], for which two parameters were defined. An expert was requested to judge the number of true spike clusters in each raw data set, before classification, and the number of identified clusters containing evidently similar and spike-like waveforms. The ratio of the latter over the first number (between 0 and 1) was used as an indication of the ability to group together true similar spikes, leaving false spikes into isolated clusters or outlier points (which could be excluded in a later off-line analysis). A second parameter was the number of unlabeled data points (i.e., spikes not assigned to any group).

#### 2.4.2. Statistical Analysis

A statistical analysis has been carried out to highlight relevant differences in the performance of the different methods on the data sets by means of Statistica (StatSoft Inc.). Each group presented as input to the statistical analysis consisted in the values of a performance index (Section 2.4.1) obtained after applying a certain method to all signals. Having assessed nonnormality of distributions (the Lilliefors test), a nonparametric multiple dependent comparison of the different groups has been performed (Friedman's test with Wilcoxon's matched pair test as* post hoc*) [42]. The significance level was established at *p* < 0.05 for Friedman's test and *p* < 0.01 for Wilcoxon's test. Data are given as median and its variation is stated as differences between 75th and 25th percentile (i.e., interquartile range, IQR).

### 2.5. Runtime Evaluation

Runtimes of the different spike sorting algorithms were compared in Matlab on the same dedicated desktop computer (quad-core 3.3 GHz CPUs with 4 GB RAM running Windows 7 64-bit). The algorithms were launched from a custom script including code for the real-time communication with a MEA A/D device (USBME-64, Multi Channel Systems GmbH), through a proprietary dynamic-link library distributed by Multi Channel Systems (“McsUsbNet.dll”). Thus, it was possible to evaluate the effective feasibility of an on-line implementation, taking into account the time required for raw data reading, filtering, spike detection, spike sorting, and storage of results. Runtimes were computed in a worst-case scenario, simulated by the occurrence of a high frequency spiking signals simultaneously in all the 60 channels (i.e., 250 Hz [44]). The evaluation was performed with a sampling frequency of 25 kHz and with varying the length of serially transmitted data blocks from the A/D device (i.e., 100 ms–3 s). Simulated signals were repeatedly loaded in the code at each new iteration. Time needed to perform the training for spike sorting (i.e., in every method apart from FSDE and O-sort) was not considered in this evaluation, supposing performance of the training off-line on a first data stream. Runtimes were computed with the Matlab functions “tic” and “toc.”. Reported data are averaged over 100 repetitions and related to one Matlab process running on one CPU (i.e., no parallel Matlab processes) [47].

## 3. Results

### 3.1. Graphical User Interface

**Figure 4:**
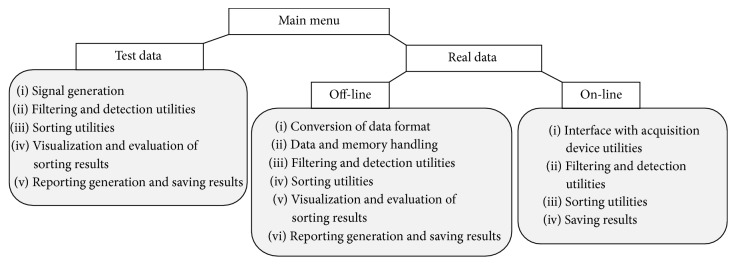
Structure and functionalities of the graphical user interface. Structure and functionalities of the GUI, which is composed of a “test data” section (intended for the analysis of simulated signals) and a “real data” section that can be used for either off-line or on-line analysis.

The algorithms described in Section 2.1 were incorporated into a GUI to speed up and ease the application and the evaluation of the methods by nonexpert users.  Figure 4 sums up the main functionalities of the GUI. The GUI is fully described in the guide provided as Supplementary Material available online at http://dx.doi.org/10.1155/2016/8416237.

**Figure 5:**
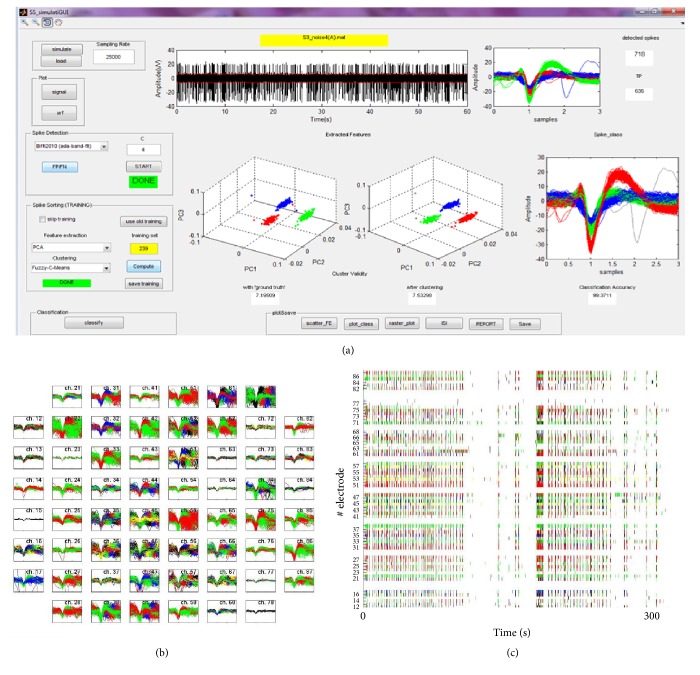
Graphical user interface. (a) Screenshot of the GUI built for spike sorting on simulated data. (b) Example of graphical result of spike sorting on multichannel MEA signals, representing the clustered spike waveforms for each electrode of the matrix. (c) Example of graphical result of spike sorting on multichannel MEA signals, representing the spike trains collected by each electrode, with spikes colored according to the signal source.

**Figure 6:**
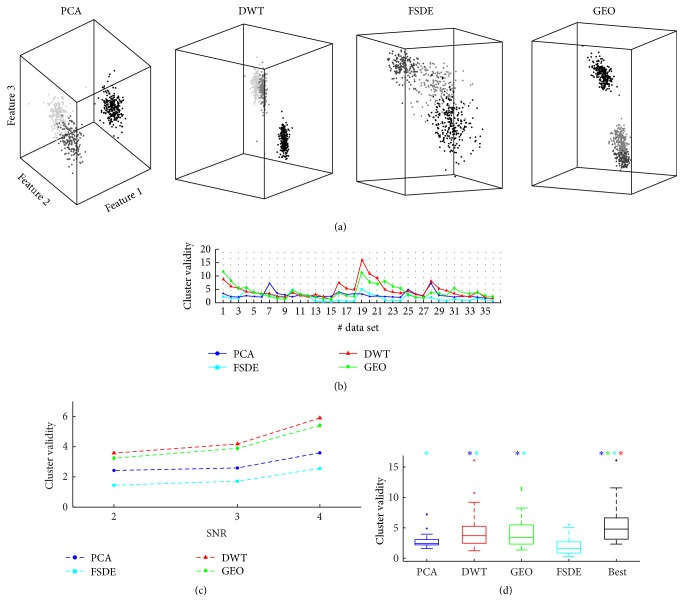
Comparison of the separability of simulated spikes in the feature space. (a) Example of projections of the spikes extracted from a simulated signal (i.e., signal #10 of Figure 2) in each feature space (PCA: Principal Components Analysis, DWT: Discrete Wavelet Transform, GEO: geometric features, and FSDE: First and Second Derivative Extrema), colored according to the real labels. (b) Cluster validity (CV) values obtained after the application of the 4 feature extraction methods to the 36 simulated extracellular signals. (c) Cluster validity dependence on different noise levels (median of CV values for each SNR group). (d) Box-plots (median and IQR with whiskers delimited by the maximum and minimum nonoutliers values) of CV values on all the simulated signals (*N* = 36). The asterisks above each method indicate statistically highest CV values of the current method compared to the method(s) coded by the asterisks' color (Wilcoxon's matched pair test with *p* < 0.01).

From a main menu (Figure 1 of Supplementary Material), two different modalities can be selected: (i) test data and (ii) real data. The “test data” interface is dedicated to the application and evaluation of spike sorting algorithms on simulated signals (Figure 2 of Supplementary Material). Specifically, the user can build a simulated signal giving as inputs the number of sources in the signals, the neurons average firing rates, and the SNR. The “real data” modality is dedicated to the analysis of real MEA data off-line (Figures 5, 6, and 7 of Supplementary Material) or on-line (Figure 10 of Supplementary Material). Both the test data and the real data “off-line” GUI allow selecting which algorithms to apply and setting all the necessary parameters for algorithm functioning (default values are the ones used in this work and reported in Section 2). After loading a signal, it is possible to perform a training on an arbitrary chosen number of spikes or to load the results of a previously performed clustering and use them for classification.

Besides manual selections of the methods, the GUI embeds an automatic routine which runs all the possible combinations of spike sorting blocks on a selected signal and displays the performance indexes (i.e., CV and CA for simulated data, ICV and CV for real data) (Figure 4 of Supplementary Material).

For a selected method, the GUI shows the performance indexes, the spikes projected and clustered in the feature space, the aligned spike waveforms, color-coded according to the clustering results, and the raster plots of each identified unit, as shown in Figure 5(a). A *∗*.txt file can be generated as a report of the analysis, together with *∗*.JPEG figures of spike sorting results (e.g., raster plots of one or multiple electrodes, grouped spike waveforms superimposed to the MEA layout, and interspike-interval histograms for each source) as in Figures 5(b) and 5(c) (see also Figures 3, 8, and 9 of Supplementary Material). The real data “on-line” GUI (Figure 10 of Supplementary Material) allows the user to select the length of data flow blocks and the sampling frequency, establishes an on-line communication with the A/D device, and performs on-line spike detection, sorting, and data storage.

The GUI and the scripts were written with Matlab R2008b, but they are compatible with all the following releases up to version R2014a. The framework is freely available upon request to alessandra.pedrocchi@polimi.it.

### 3.2. Spike Sorting Accuracy

#### 3.2.1. Simulated Data Sets

*(1) Feature Extraction*.  Figure 6(a) shows an example of the projection of simulated neuronal spikes on different feature spaces (i.e., PCA, DWT, GEO, and FSDE). Concerning the DWT-based feature extraction method, DWT with 3, 4, or 5 decomposition levels was performed and the feature extraction performance in terms of cluster validity (CV) was evaluated projecting 3, 4, or 5 coefficients in the feature space. Since three and four decomposition levels provided statistically highest CV values, the three-level DWT algorithm has been selected to keep a lower complexity. The same consideration led us to the selection of a 3D feature space for the GEO method. Thus, results reported here about DWT and GEO method were obtained with the above-mentioned settings.

The FE effectiveness assessed on each signal with the different feature extraction methods is shown in Figure 6(b). Overall, the DWT method yielded the best performance on 12 signals (including 5 signals with SNR = 4, 3 signals with SNR = 3, and 4 signals with SNR = 2). Also the GEO method provided good results at different noise levels, being the best method on 14 signals including 4 with SNR = 4, 6 with SNR = 3, and 4 with SNR = 2. The PCA yielded the best separability for 9 signals (2 with SNR = 4, 3 with SNR = 3, and 4 with SNR = 2). Finally, the FSDE method yielded the poorest performance, being susceptible to the noise corrupting the extracted waveforms. But it was the best solution for 1 high SNR signal (i.e., signal #10).

All the methods showed a similar trend of reduced performance when the noise level was increased (Figure 6(c)). In particular, CV values yielded by PCA decreased by 30% when the SNR was lowered from 4 to 2, while CV values yielded by the other FE methods decreased by 40%.

For PCA, DWT, and FSDE FE methods, the waveforms similarity index (see Section 2.2) was not a distinguishing factor to obtain a good separability. This parameter yielded a visible impact on the GEO method, whose CV values dropped in cases of high waveforms similarity (e.g., signals #7-8-9 and #25-26-27).

Overall, the DWT and the GEO feature selection yielded a comparable CV (*p* > 0.01), higher than the temporal PCA feature selection and the FSDE method (*p* < 0.01), as shown in Figure 6(d). The application of the most effective feature extraction method for each signal resulted in the statistically highest CV as compared to the application of the same method to all the signals (Figure 6(d), black box-plot).

**Figure 7:**
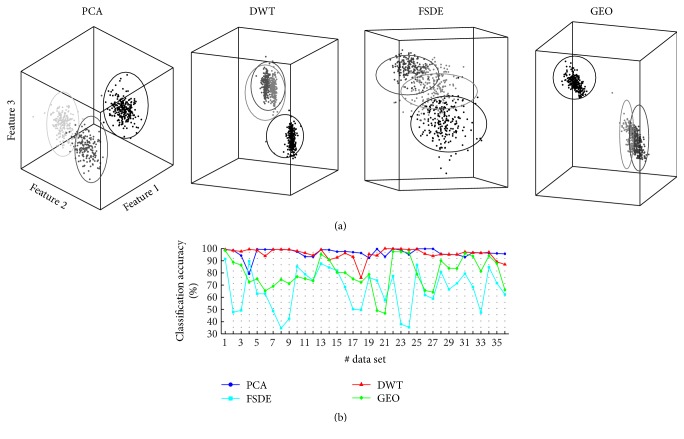
Comparison of classification accuracy on the simulate data sets with the benchmark *K*-means method. (a) Example of projections of the same data set (i.e., number 10 of Figure 2) in each FE space and results of *K*-means clustering of the features. Spikes are colored according to the real label. Superimposed circles are the clusters found by *K*-means. (b) Classification accuracy values for the 36 simulated signals.

*(2) Clustering*. Besides the specific clustering algorithm, the accuracy of a clustering method depends on the separability of features and their distribution/shape in the feature space. To first focus on the effect of the previous FE step, all the considered FE algorithms were coupled to the benchmark *K*-means clustering and then compared. As an example of this, Figure 7(a) illustrates the classification performed by *K*-means on the same data set of Figure 6(a) in each of the feature spaces.  Figure 7(b) shows the trend of classification accuracy (CA) yielded by *K*-means coupled to the 4 FE methods across all the simulated data sets. *K*-means coupled to both DWT and PCA achieved statistically the highest and comparable classification performances (*p* > 0.01). The high accuracy of DWT+*K*-means combination is ascribable directly to the high separability provided by DWT (Figure 6(d)). Even if PCA did not provide the best CV values (Figure 6(d)), the high accuracy of the PCA+*K*-means combination can be explained by the almost spherical shapes of clusters in the feature space (e.g., Figure 7(a)), which best matches the *K*-means classification. On the contrary, *K*-means clustering applied on GEO features yielded poor accuracy, most likely because the clouds of spikes created in the GEO feature spaces, even if compact (Figure 6), have no spherical shapes (e.g., in Figure 7(a)). Clustering of FSDE features provided the worst results when compared to any other method, due to the poor separability already shown in Figure 6.

As mentioned earlier, part of the results shown in Figure 7 depends on the specific properties of the *K*-means clustering. We have compared the classification accuracy of all the tested FE methods combined with different clustering methods, and obtained the performances shown in Table 4.

The performances of FCM clustering are shown with respect to two different degrees of fuzziness (i.e., *m* = 1.1 and *m* = 3). Overall, FCM with *m* = 3 provided statistically higher or comparable accuracy compared to FCM with *m* = 1 (i.e., a value which makes FCM similar to the *K*-means method [43]). The performances of the DBC method are reported in the case of two different resolution levels of the 3D-LUT (i.e., *N* = 16 and *N* = 32) and of resolution *N* = 32 with an additional automatic cluster merging step. The highest accuracy levels were obtained in the third configuration. The lower resolution of the density map led also to acceptable clustering, but very close clusters could not be distinguished. The worst performance was obtained building the LUT with *N* = 32 without the cluster merging step. In this case the outcome is affected by overclustering, since a lot of little clusters were identified.

Results of the statistical comparison of clustering methods applied after a given FE method are presented in Table 4. After PCA, the application of DBC or FCM provided an accuracy comparable to the benchmark *K*-means clustering (median CA > 97%). Concerning DWT, the combination with FCM yielded accuracy values comparable to *K*-means (median CA > 95%). GEO features achieved the best results (median CA > 88%, statistically higher than CA achieved with *K*-means) if coupled to DBC, which is suitable to identify the clusters even if they have no spherical shapes. Only the FSDE method could not reach satisfactory accuracy with any of the clustering methods (median CA < 70%) on our data.

**Figure 8:**
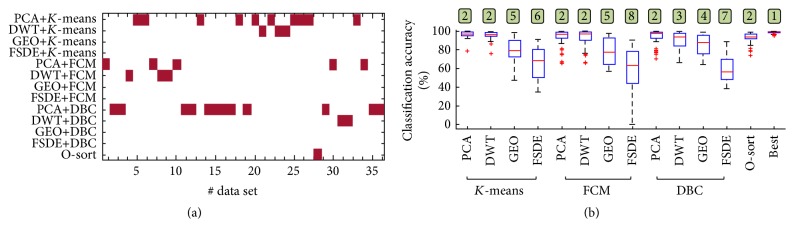
Classification accuracy on the simulated data sets. (a) Indication of which method yielded the highest classification accuracy (CA) for each data set (marked by the red box). (b) Box-plots (median and IQR with whiskers delimited by the maximum and minimum nonoutliers values) of classification accuracy provided by all the methods on all the data sets (*N* = 36). The statistically significant differences are indicated as the numbers above each box-plot, the box-plot being marked with “1” referring to the method with highest CA compared to all the others and the box-plot marked with “8” referred to the method with the lowest CA compared to all the others (Friedman's test followed by Wilcoxon's matched pair test, *p* < 0.01).

The statistical analysis applied to all the possible combinations of methods confirmed the absence of a unique method outperforming the other when applied indiscriminately to all the signals. Indeed, PCA+*K*-means, PCA+FCM, DWT+*K*-means, DWT+FCM, PCA+DBC, and O-sort yielded a comparable accuracy (Figure 8). However, these performances were statistically lower compared to the utilization of the best clusterer for each signal (Figure 8(a)), as illustrated by box-plots in Figure 8(b) (box-plot on the right).

#### 3.2.2. Real Data Sets

**Figure 9:**
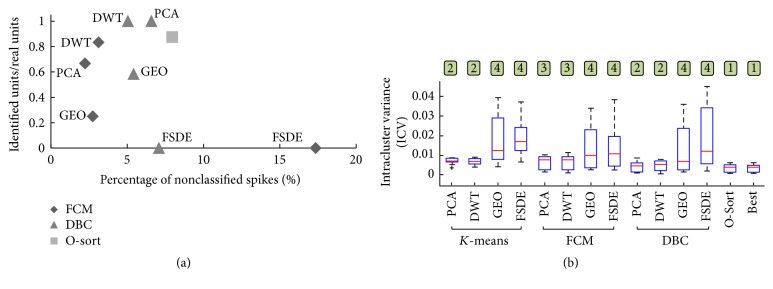
Performances of the methods on real data. (a) Outcome of the visual inspection on the results of the methods, where the percentage of nonclassified spikes and the ratio between the number of correctly identified clusters and the real number of clusters are reported. Each symbol represents a combination of algorithms, as indicated by the legend and annotations in the graph. *K*-means is not represented since it does not provide unclassified spikes. (b) Box-plots (median and IQR with whiskers delimited by the maximum and minimum nonoutliers values) of the intracluster variance (ICV) for each of the FE and clustering combinations and O-sort applied to all the real signals (*N* = 10). The statistically significant differences are indicated as the numbers above each box-plot, the box-plot being marked with “1” referring to the methods with lowest ICV compared to all the others and the box-plot marked with “4” referred to the methods with highest ICV compared to all the others (Friedman's test followed by Wilcoxon's matched pair test, *p* < 0.01).

Spike sorting performances measured on real data by visual inspection and by quantitative assessment (i.e., intracluster variance, ICV) were proven to be in good agreement with results on simulated signals. For each combination of FE and clustering algorithms and O-sort, Figure 9(a) shows the scatter plot of the percentage of unclassified spikes and the ratio between the number of identified clusters containing evidently similar and spike-like waveforms and the true number of clusters (mean values across *N* = 10 signals). DWT and PCA features, combined with both FCM and DBC, provided the best performances, since they lie in the upper left area of the graph. GEO features of real spikes combined to either DBC or FCM performed worse than PCA and DWT. Finally, cluster methods combined with FSDE features could not correctly identify almost any cluster, mostly because real spikes and noise were merged inside poorly compact clusters. The figure also shows that DBC left a higher number of unclassified spikes than FCM. Indeed, if the shape of the spikes changes after the definition of the LUT, due to bursting events or an increase of the noise [48], the incoming spikes could be not labeled at all. Also O-sort provided a high percentage of unclassified spikes, due to the tendency to overclustering (i.e., creating many small clusters). As shown in Figure 9(b), *K*-means and DBC clusterers coupled to PCA and DWT yielded a statistically lower ICV than FCM, but higher than O-sort, which created the most compact clusters, comparable to the application of the best method for each signal. FSDE and GEO coupled to any clusterers resulted in the worse (i.e., highest) ICV.

### 3.3. Spike Sorting Runtimes

An evaluation aimed at comparing Matlab execution time relative to the feature extraction and clustering steps was performed. Parameter values set for this evaluation were the ones which allowed the best performance for each method (see Section 3.2).  Table 5 reports the number of operations (i.e., additions, multiplications, and if-operations) required to process on-line a single spike in the implemented Matlab code and the resulting runtimes in Matlab.

Extraction of features from each input spike takes on average a comparable time for PCA, FSDE, and GEO methods (i.e., 5-6 *μ*s). In spite of the higher number of additions and multiplication compared to the other methods, PCA is time effective thanks to the absence of comparison operations. The DWT-based method requires a time one order of magnitude higher (i.e., 64 *μ*s) because of the significantly highest number of operations.

FCM classification of one spike is fourfold slower than DBC classification (i.e., 27 *μ*s versus 7 *μ*s). This difference is ascribable to the fact that DBC requires only the consultation of a look-up-table. O-sort algorithm takes a higher time (on average 210 *μ*s per spike), since its implementation involves not only arithmetic operations but also large amounts of memory and control logic.

### 3.4. On-Line Feasibility Evaluation

**Figure 10:**
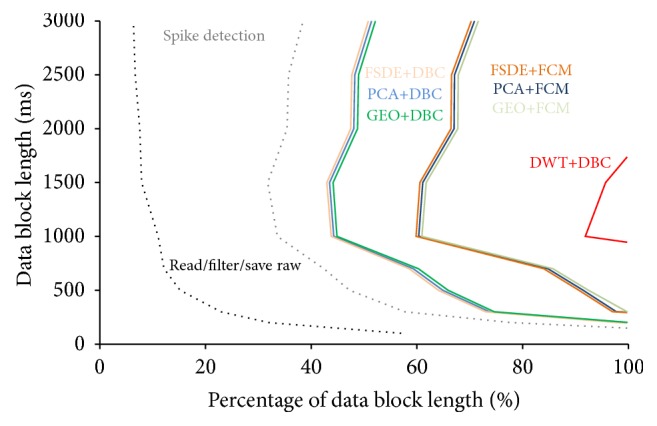
Evaluation of runtimes of the spike sorting algorithms. Runtimes measured in the experimental setup, for different lengths of input data block (ms) sent from the acquisition device to Matlab. Runtimes were measured in a worst-case scenario of high firing activity simultaneously occurring at all the 64 channels. Values related to raw data reading, filtering, spike detection, and classification with all the possible methods are reported. The runtime is related to input data block length (i.e., the time available for processing before the buffer update) and is expressed as its percentage (e.g., a runtime percentage equal to 60% for a 1 second block means that there is a margin of 400 ms for further operations). Times are for Matlab running on a quad-core 3.3 GHz CPUs desktop computer with 4 GB RAM and Windows 7 64-bit.

The experimental test performed with the setup described in Section 2.5 showed that an on-line processing of MEA signals is possible using the implemented framework. To allow that, the Matlab execution time spent to process a data block containing 64-channel raw data should be lower than the length of serially transmitted data blocks from the A/D device. The most time effective coding turned out to be a sequential processing over the channels. Given as input data buffer, each channel is filtered before being scanned for spikes; then each detected spike is given as input to the feature extractor and classifier.  Figure 10 reports the runtimes for the processing of 64 channels in a worst-case input scenario (i.e., 1 spike every 4 ms at every channel). The runtime is related to input data block length (i.e., the time available for any processing before the buffer update) and is expressed as its percentage (e.g., a runtime percentage equal to 60% for a 1-second block means that there is a margin of 400 ms for further operations). Runtimes include the time for raw data reading, filtering, and saving (gray dashed line), plus spike detection (black dashed line), plus feature extraction and clustering (lines colored according to the combinations of the algorithms). In the example, reported spike detection runtime relates to a negative threshold-based spike detection method. Generally, “AdaBandFlt” takes more time (i.e., up to 4-fold in the case of maximum data block length), due to its higher complexity, suggesting that further optimization is needed.

In the simulated scenario of high spiking frequency simultaneously in all the channels, all the feature extraction methods (apart from DWT), coupled to both clustering methods, can process data before buffer overwriting, if the data block length is higher than ~300 ms. When using shorter data blocks, the operation of raw data reading from the acquisition device (black dashed line in Figure 10) takes the highest percentage of the available time, so that the only feasible processing operation is spike detection (gray dashed line). When using data blocks as long as 1 second, the runtime of all the methods (except DWT) is lower than 600 ms (i.e., 60% of data block length), indicating that further operations are feasible. As expected from evaluations on single-spike runtimes, the DWT-based feature extraction, although highly accurate in many cases, is hard to be performed on-line in the actual implementation. The tests showed that, only in the case of data block length between ~700 and ~1500 ms, a classification with DWT coupled to DBC was possible in the worst-case scenario but leaving a poor margin (lower than 10%) for additional operations. Finally, the O-sort method could not run on-line in the proposed implementation, as expected from results on single-spike runtimes reported in Table 5.

## 4. Discussion and Conclusions

### 4.1. Usefulness of the Present Work

The present work addressed two important issues in the field of spike sorting of neuronal signals collected by means of MEAs, which are the very limited availability of data-centric and on-line spike sorting tools. Our aim was to provide a framework for an easy comparison of different spike sorting algorithms on the same data which would be suitable for off-line and on-line data analysis. Rather than proposing a new sophisticated algorithm, we exploited the modularity of existing spike sorting processes, that is, the presence of several steps and different techniques that can be mixed and matched to adjust the process to the data set. Therefore, the implemented toolbox integrates different spike sorting blocks (i.e., four feature extraction methods, three feature clustering methods, and one template matching clustering), which have been selected from the literature. Thus, it provides the possibility to choose the algorithm that optimally performs on a specific channel data most suitably, in contrast to commonly used tools which apply one predefined method to all electrodes. The pool of algorithms integrated in the framework presents features of automaticity and simplicity that are important requirements in spike sorting and facilitate on-line implementations [31].

Besides the modular software tool, the work has presented an extensive evaluation of the different combinations of feature extraction and clustering methods integrated in the framework. In order to help the users in the selection of their best methods for data processing and to guide the evaluation of multiple algorithms on other data sets, the algorithms were tested both on simulated data sets (as most commonly done to assess the performance of spike sorting methods [13, 20]) and on real multiunit recordings from neuronal dissociated cultures, that is, in more realistic conditions [24, 46]. As expected, the selection of channel-centric methods (i.e., the best method for each channel) provided the statistically highest accuracies level. These results support the usefulness of different spike sorting options in the same framework. Furthermore, the realized framework lends itself to be exploited also to explore possible associations between signal features (e.g., waveforms similarity and SNR value) and the accuracy of spike sorting methods.

### 4.2. Advantages of the Matlab Framework for Spike Sorting

Matlab has been primary chosen because the MEA users often share algorithms written in Matlab language, which can facilitate the utilization and extension of the framework. Despite existing open-source alternatives (e.g., Python), Matlab is still a very common framework for neurophysiologists and research institutions working with MEAs [49, 50]. The spike sorting codes implemented in this work could thus be easily tested and incorporated into existing Matlab-based frameworks offered by the community and dedicated to different kinds of analysis of MEA data (e.g., “Spycode” [45], “Find” [51], “Wave_clus” [20], “NeuroQuest” [31], “DrCell” [34], “SigMate” [50], “QSpike tools” [52], and “Manta” [32]).

For running in the off-line mode, the GUI integrates the Neuroshare API library, which is a community-supported vendor-neutral library. Therefore, it would be possible to import into Matlab neural data files acquired by different platforms from the one we used (Multi Channel Systems GmbH) with minor modifications. For running in the on-line mode, actually two types of acquisition boards from Multi Channel Systems were tested (i.e., USB-ME64/128 and MEA2100). However, the framework is expected to be compatible with other acquisition boards provided that an* ad hoc* communication interface is built in Matlab. This would be easily performed exploiting the Data Acquisition toolbox of Matlab, which allows acquiring data from a variety of DAQ hardware [32].

### 4.3. Discussion of On-Line Spike Sorting Operations

Taking into consideration that Matlab is slower in performing some operations compared to low level languages [52] and thus it is not the best choice for real-time analysis (e.g., closed-loop MEA experiments), we explored its potentiality to run MEA data classification (including also the preceding filtering and spike detection) during on-line open-loop recordings. We demonstrated that the realized implementation (60 channels) was able to respect the time constraints running most of the evaluated spike sorting algorithms in a worst-case realistic scenario, where simultaneously a high firing activity is collected by all the channels. The runtime evaluation took into account also times for accessing data sent by the A/D device that we use in our lab and showed that a good temporal margin is achievable with data blocks length longer than 500 ms. However, considerations about the length of data blocks reported in Section 3 can be generalized to any A/D board which sends dataflow to the acquisition computer by means of data blocks. Clearly, a much lower data block length would reduce the delay between acquisition and processing, but this issue is not relevant when the aim is to speed up analysis of open-loop recordings. Moreover, a too short data buffer would increase the probability of splitting spike waveforms or burst events into two different time epochs.

### 4.4. Future Work

Further code optimization activity will be performed to reduce the time for spike sorting blocks currently requiring too long computational runtime. To this aim, a more careful optimization of codes, an implementation of MEX-files of all the spike processing steps, and the resorting to the Parallel Computing toolbox [49] will reveal the easiest options to provide improvements over the numbers reported here. If this is not suitable, we plan to exploit the Parallel Computing toolbox to access the graphical processing unit (GPU) of the computer [34, 53].

Moreover, an issue of the current implementation is that on-line parallel data visualization is not possible, due to speed limits of Matlab graphics [32]. Possible solutions could be to test a range of efficient coding strategies to speed up data plotting [32] or to integrate a plotting plugin written in a faster GUI programming language such as C# [31, 35].

Besides improvements centered on shortening algorithm runtimes, a future activity will be focused on the automatization of the training phase. To this aim, an evaluation of the time needed to train the data (e.g., acquire a data fragment until a minimum number of spikes have been detected) and the most reasonable frequency of the training during acquisition will be performed. A repetition of the training would be preferred especially for long-lasting experiments since (i) only neurons which fire during the learning phase can be classified and (ii) the physical relations between neurons and electrodes may change due to cell growth (nonstationarity). A possible solution could be to perform a training step whenever a metric referring to the goodness of clustering detects the fact that data have changed their features to a considerable extent, as suggested by a recent work [30].

Furthermore, a possible enrichment of the tool would be to provide the possibility to combine heterogeneous features extracted from the spikes (e.g., DWT, PCA, and GEO) allowing taking advantage of the strengths of each feature extraction method to achieve better performances [4]. This implementation would likely require a longer time for the initial training phase and, during on-line mode, the computation of several features within the time constraints. Thus, it will be incorporated after the afore-mentioned optimization steps. Finally, the integration of methods to resolve the issue of spike overlapping [19, 54] may be considered.

## Supplementary Material

The supplementary material includes the description of the Matlab toolbox for neuronal spike sorting presented and validated in the work (see Results 1.1). In particular, guidelines on how to use the ‘test data' GUI as well as the ‘real data' off-line and on-line GUIs are here provided together with graphical examples.

## Figures and Tables

**Table 1:** Overview of spike sorting algorithms.

Reference	Feature extraction	Clustering
Letelier and Weber [14]	Wavelet	Fuzzy-*C*-means
Harris et al. [15]^a^	PCA^c^	Expectation maximization
Zouridakis and Tam [16]	Waveforms	Fuzzy-*C*-means
Hulata et al. [17]	Wavelet	*K*-means
Egert et al. [18]^a^	PCA	Manual cluster cutting
Shoham et al. [19]	PCA	Expectation maximization
Quiroga et al. [20]^a^	Wavelet packet coefficients	Superparamagnetic clustering
Rutishauser et al. [11]^a^	—	Template matching
Cho et al. [21]	LDA^d^	Fuzzy-*C*-means
Adamos et al. [22]	PCA	Expectation maximization
Awais and Andrew [23]	Zero crossing	*K*-means
Biffi et al. [24]	PCA	Hierarchical clustering
Takekawa et al. [25]	Wavelet	Bayes
Gibson et al. [13]	Discrete derivative	Fuzzy-*C*-means
Cheng et al. [26, 27]	PCA	Density-based clustering
Liu et al. [28]	PCA	Valley-seeking
Lai et al. [29]	Wavelet	Gray relation analysis
Bestel et al. [4]	PCA, wavelet, geometrical features	Expectation maximization
Yuan et al. [12]	Wavelet	*K*-means, template matching
Oliynyk et al. [30]^a^	PCA	Fuzzy-*C*-means^f^
Kwon et al. [31]^a^	DWT^e^, PCA, peak-to-peak	Expectation maximization, *K*-means, fuzzy-*C*-means, manual cluster cutting
Englitz et al. [32]^a^	Geometrical features	1D clustering^f^
Paraskevopoulou et al. [33]	FSDE	*K*-means
Nick et al. [34]^a^	PCA, DWT, geometrical features	Expectation maximization
MCRack (Multi Channel Systems GmbH)^b^	—	Manual amplitude window^f^
Spike2 (Cambridge Electronic Design Ltd.)^b^	PCA	Template matching^f^ Manual cluster-cutting, *K*-means, Gaussian mixture models
Off-Line Sorter (Plexon Inc.)^b^	PCA	Expectation maximization, *K*-means, valley-seeking

Overview of the literature about spike sorting algorithms, including published papers about methods, custom toolboxes, and commercial software. ^a^Custom toolbox available to the community. ^b^Software coupled to a commercial acquisition platform. ^c^Principal component analysis. ^d^Linear Discriminant Analysis. ^e^Discrete Wavelet Transform. ^f^On-line mode. Keywords used for literature search were “spike sorting”, “spike detection”, “spike classification”, “*in vitro*”, “real time/on-line”, “Microelectrode arrays”, and “toolbox” (PubMed and Google Scholar).

**Table 2:** Properties of the selected feature extraction methods.

	Domain	Percentage of publications (with respect to Table 1)	Need of training before on-line FE
Principal Components Analysis (PCA)	Time	36%	Yes
First and Second Derivative Extrema (FSDE)	Time	3%	No
Geometric features (GEO)	Time	13%	Yes
Discrete Wavelet Transform (DWT)	Time/scale	26%	Yes
Other methods	—	22%	—

The “domain” column refers to the analysis domain in which each method works, for example, time domain or time/scale domain. The “percentage of publications (with respect to Table 1)” is the ratio between the number of publications dealing with a certain FE method and the total number of analyzed publications (reported in Table 1). To esteem this parameter, works dealing with *N* methods were counted as *N* different works in the denominator. Different works of the same authors using the same FE method were counted as 1. The “need of training before on-line FE” column indicates whether a preliminary training phase on a first set of acquired spikes is needed in order to run the method in on-line mode.

**Table 3:** Properties of the implemented and evaluated clustering algorithms.

	Input	Percentage of publications (with respect to Table 1)	Automaticity	Parametric	Need of training before on-line clustering
*K*-means	Spike features	30%	Yes^a^	Yes	—
Fuzzy-*C*-means (FCM)	Spike features	25%	Yes^a^	Yes	Yes
Density-based (DBC)	Spike features	4%	Yes	No	Yes
O-sort	Spikes	11%	Yes	Yes	No
Other methods^b^	—	30%	—	—	—

The “percentage of publications (with respect to Table 1)” is the ratio between the number of publications dealing with a certain clustering method and the total number of analyzed publications (reported in Table 1). “Automaticity” refers to the possibility not to define a number of clusters a priori. “Parametric” refers to the need to set one or more threshold values for parameters involved in the algorithm. The “need of training before on-line clustering” column refers to the necessity of an off-line phase to train the algorithms on data before an on-line classification can be performed. ^a^The optimization of a proper index during training phase is needed. ^b^Including nonautomatic methods (e.g., manual methods) and methods not suitable for on-line mode.

**Table 4:** Classification accuracy of all the tested methods.

	*K*-means	FCM		DBC			O-sort
		*m* = 1.1	*m* = 3	*N* = 16	*N* = 32	*N* = 32 automatic merging	
PCA	97.16^*∗*^ (4.09)	98.46^*∗*^ (9.64)	97.11^*∗*^ (6.45)	94.76 (12.15)	91.04 (27.45)	97.66^*∗*^ (5.92)	—
DWT	96.42^*∗*^ (4.78)	77.18 (31.08)	95.29^*∗*^ (8.81)	93.60 (12.68)	76.13 (24.47)	93.90 (13.39)	—
FSDE	68.42 (29.64)	69.83 (14.07)	63.66 (33.26)	67.01 (25.22)	36.97 (50.62)	56.71 (21.24)	—
GEO	79.57 (17.82)	72.25 (28.26)	77.43 (28.63)	82.35 (19.77)	84.99 (18.29)	88.10^*∗*^ (20.04)	—
	—	—	—	—	—	—	94.37 (4.75)

Spike sorting classification accuracy, CA (%), on the simulated data sets for all the possible combinations of FE (rows) and clustering algorithms (columns) and for O-sort. CA is presented as median and (IQR) over the different signals (*N* = 36). For each FE method (i.e., first 4 rows of the table) the asterisk points out the clustering method with the statistically highest CA performance (*p* < 0.01, the Friedman + Wilcoxon test). Accuracies lower than 70% (e.g., FSDE method) were not considered acceptable.

**Table 5:** Computational effort and runtime to process a single spike.

Method	Number of additions	Number of multiplications	Number of if-operations	Time (*μ*s)
PCA^*∗*a^	3*n* ^e^	3*n*	0	5.8 ± 3.3
DWT^*∗*b^	4*n*	6*n*	0	64 ± 33
FSDE^*∗*^	2*n* − 3	0	2(*n* − 1)	5.2 ± 4.3
GEO^*∗*c^	2*n*	1	6*n* − 2	6.5 ± 3.9
FCM	5*C* ^f^	6*C*	*C*	27 ± 0.6
DBC	0	0	6*N* ^g^	7.3 ± 0.2
O-sort^d^	6*Cn* + 2*n*	6*Cn* + 2*n* + 1	8	210 ± 200

Computational requirements to classify a single spike (3 ms waveform sampled at 25 kHz). Columns from 2 to 4 indicate the number of operations for each spike included into the implemented Matlab code, showing their dependence on algorithms parameters. The last column reports the resulting execution times (averaged over 100 repetitions and reported as mean ± standard deviation). Times are for Matlab running on a quad-core 3.3 GHz CPUs desktop computer with 4 GB RAM and Windows 7 64-bit. Asterisks in the first column indicate methods for which an implementation in C language with MEX-files was performed.

^a^Projection onto 3 principal components.

^b^3-level wavelet decomposition.

^c^Extraction of 7 geometric features from the spike waveform.

^d^Worse case in which all the cycles/iterations involved in O-sort method are entered.

^e^ *n*: number of samples per spike (*n* = 75 in the numerical time example).

^f^ *C*: number of clusters or templates (*C* = 4 in the numerical time example).

^g^ *N*: resolution of the look-up-table for density-based clustering method (*N* = 32 in the numerical time example).
