# Supplementary material for: A Framework for the Comparative Assessment of Neuronal Spike Sorting Algorithms towards More Accurate Off-Line and On-Line Microelectrode Arrays Data Analysis
Source: Comput Intell Neurosci. 2016 Apr 27;2016:8416237. doi: 10.1155/2016/8416237 (PMC4863096; doi:10.1155/2016/8416237)
Supplement: Supplementary file 1 — The supplementary material includes the description of the Matlab toolbox for neuronal spike sorting presented and validated in the work (see Results 1.1). In particular, guidelines on how to use the ‘test data' GUI as well as the ‘real data' off-line and on-line GUIs are here provided together with graphical examples. [file 8416237.f1.pdf]

Supplemental material for:

A framework for the comparative assessment of neuronal spike sorting algorithms towards more accurate off-line and on-line Microelectrode Arrays data analysis

Giulia Regalia[1*], Stefania Coelli[1], Emilia Biffi[1#], Giancarlo Ferrigno[1], Alessandra Pedrocchi[1]

[1] Neuroengineering and medical robotics laboratory, Department of Electronics, Information and Bioengineering, Politecnico di Milano, Milano, Italy

* Corresponding author

E-mail: giulia.regalia@polimi.it

Currently working at the Bioengineering Laboratory, Scientific Institute IRCCS Eugenio Medea, Bosisio Parini, Italy

General Information about the Toolbox:

The toolbox was implemented in MATLAB : version 7.7.0.471(R2008b) [The Mathworks.Inc] and it is compatible up to version R2014a.

Its main aim is to provide a Graphical User Interface (GUI) to help an interested user in analyzing its own MEA data through our framework of algorithms.

The toolbox is composed by several GUIs and MATLAB (I/O) functions.

These are organized in folders as follow:

1. ***function_for_detection&sorting*** : contains MATLAB .m and .mex functions for the spike sorting algorithms. They are divided in modules and the can be used independently of the GUI.
2. ***GUI_testdata***: contains the functions and the GUI to test the algorithms on single simulated signals.
3. ***GUI_offline***: contains the functions and the GUI figure for the offline analysis on real single or 60-channels MEA signals.
4. ***GUI_online*** : contains a script which implements the real-time communication between Matlab and an A/D MEA device from Multi Channel Systems GmbH and applies on-line spike-detection and sorting.
5. ***nsMCDLibrary_3.7b***: Neuroshare API library to convert from proprietary MEA data format to Matlab format (downloaded at: http://neuroshare.sourceforge.net/).
6. ***McsUsbNet.dll*** : .NET proprietary dynamic-link library distributed by Multi Channel Systems GmbH to access MCS devices from Matlab code (downloaded at http://www.multichannelsystems.com/software/mcsusbnetdll).
7. ***wf_example***: .mat files containing sample spike waveforms (from hippocampal and cortical cultures) and noise templates used to simulate MEA signals.
8. ***test_data_set.zip***: zip archive containing 36 simulated MEA signals using the traces contained in the folder "wf_example".
9. ***mcd_example.zip***: zip archive containing a sample 60-channel MEA recording performed with a A/D device from Multi Channel Systems GmbH (USB-ME64).

Note: despite .mex and the relative .c source codes are provided, they are not actually used in the GUIs codes because their compatibility with the users' MATLAB cannot be guaranteed.

Thus in the codes there are the relative MATLAB versions and the commands referred to mex-files are commented. In this way the user can decide if enable the MEX_file (a compatible compiler may be necessary, see www.mathworks.com), which is especially recommended to speed up on-line analysis.

Installation:

After receiving the .zip folder containing the toolbox unzip it and add all the folders to your MATLAB path. This should provide all the functionalities to work fine.

How to Start:

In the MATLAB command window type:

>> Main_GUI

The Main_GUI will pop-up and show three buttons (Fig.1) :

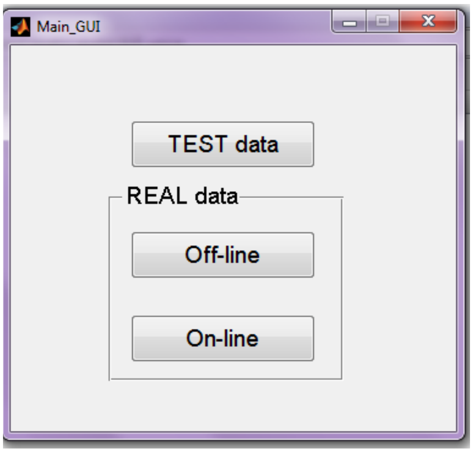

Figure 1: Main menu GUI

The three keys stand for:

1. 'TEST data' opens the Spike sorting GUI for simulated signals analysis.
2. 'REAL data'
    a. Off-line: opens the Spike sorting GUI for real multi-channel signal analysis off-line
    b. On-line: opens the Spike sorting GUI for real multi-channel signal analysis on-line.

‘TEST data’ : Spike sorting GUI for simulated signals

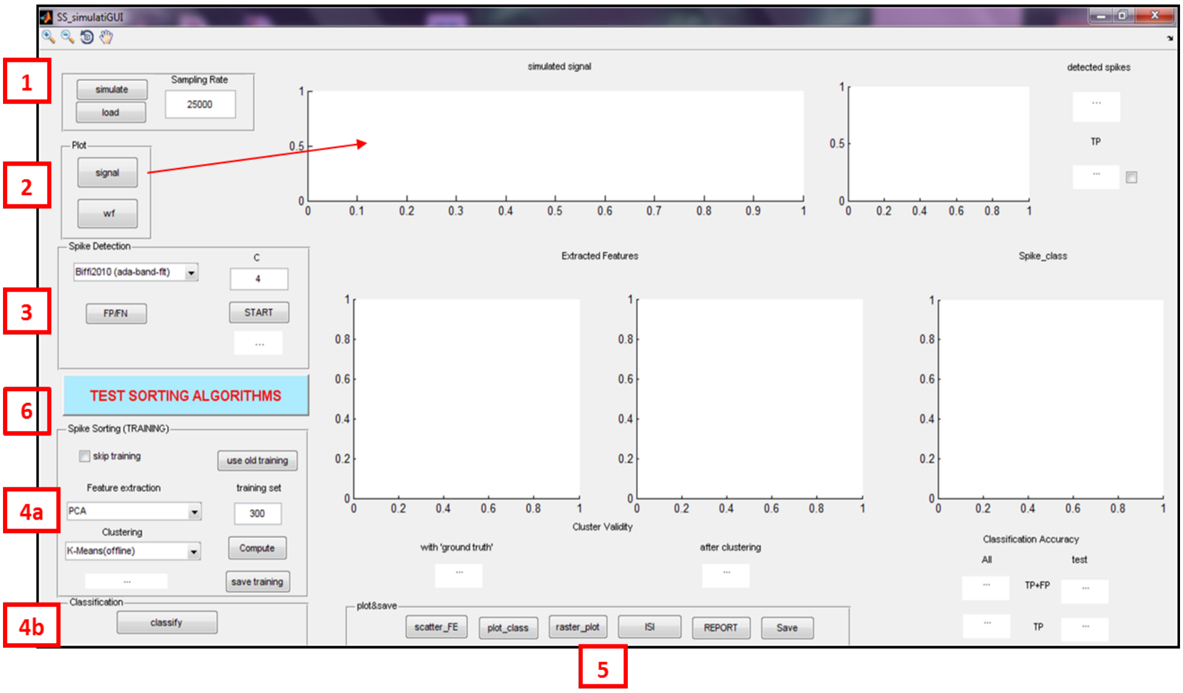

Figure 2: The GUI figure for Spike Sorting for simulated signals analysis

This GUI (Fig. 2) allows the user to load and analyze simulated spike data. Signals can also be created and then loaded **[1].** The simulation needs some input parameters that the user has to provide in the command window :

1. sampling rate
2. number of neurons,
3. firing rate for each unit
4. signal length in seconds

In the same panel the sampling rate has to be given in input (default =25000 Hz).

The loaded signal can be plotted through the command ‘**signal**’ in **[2].**

In ‘Spike Detection’ **[3]**, the user can choose the detection algorithm (AdaBandFlt or MCR) and the threshold factor C. When the text box under the button ‘**start**’ turns green, it is possible to plot the detected waveforms (command ‘**wf**’ in **[2])** and check the detection accuracy (CA) through the button **FP/FN**. The results are shown near the plotted spikes.

[4a] is dedicated to spike sorting (training). Users can select the amount of spikes for the training (default =1/3 of the extracted spikes), the feature extraction algorithm and the clustering method through the dedicated menu.

'**Compute**' starts the training phase.

When the training is ready the text-box under the clustering menu turns yellow), and the user has to click the button in **[4b]**.

Notes:

-If K-means is selected in the 'Clustering' menu, the text box turns green and the sorting is finished, because there is not a classification phase on test new spikes.

-The 'automatic selection' option in the 'Features extraction' menu computes all the different features and automatically select the best three according to the 'Maximum difference' algorithm (see paragraph 2.1.2.2 of the Manuscript). *The current version is feasible only in off-line mode.*

'**save training**' allows to save all the parameters and the results about the training phase if it is not possible to perform the classification immediately, therefore in a following session on the same data all the information is saved and can be loaded (skip training→ use old training).

[5] provides some graphical commands:

Scatter_FE can be used right after the selection of the FE method and display the training spike in the feature space.

Plot_class allows the visualization of the spikes colored with the 'ground truth' label and the spike sorting results in the feature space and the spike waveforms colored with the found classes. The value of cluster validity index and classification accuracy are calculated and displayed.

Raster_plot shows the raster plot for each neuron sorted

ISI shows the inter-spike interval histograms for each single unit sorted.

Report creates a .txt file and a .jpeg figure with the main information of the analysis (Fig. 3).

Finally, the 'TEST SORTING ALGORITHMS' button [6] provides an automatic comparison of all the possible combination of FE and clustering methods. The number of decomposition levels for the wavelet algorithm and the quantization level for the DCB clustering method are to be given in advance. Results are displayed in a Figure (Fig. 4).

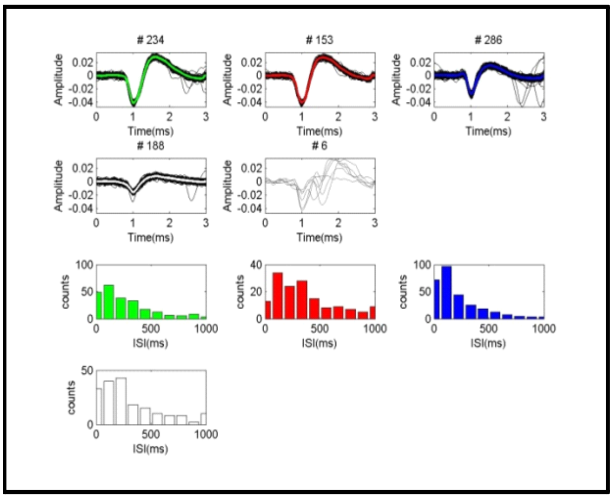

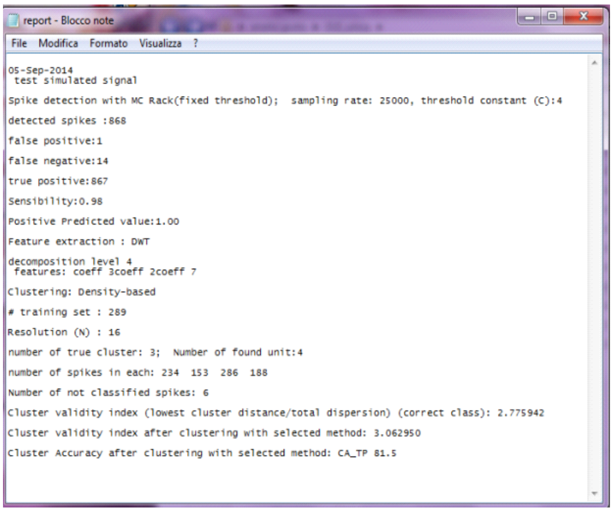

report - Blocco note

File Modifica Formato Visualizza ?

```
05-Sep-2014
 test simulated signal

Spike detection with MC Rack(fixed threshold);  sampling rate: 25000, threshold constant (C):4

detected spikes :868

false positive:1

false negative:14

true positive:867

Sensibility:0.98

Positive Predicted value:1.00

Feature extraction : DWT

decomposition level 4
 features: coeff 3coeff 2coeff 7

Clustering: Density-based

# training set : 289

Resolution (N) : 16

number of true cluster: 3;  Number of found unit:4

number of spikes in each: 234  153  286  188

Number of not classified spikes: 6

Cluster validity index (lowest cluster distance/total dispersion) (correct class): 2.775942

Cluster validity index after clustering with selected method: 3.062950

Cluster Accuracy after clustering with selected method: CA_TP 81.5
```

Figure 3: the final report .jpeg figure and an example of .txt report file

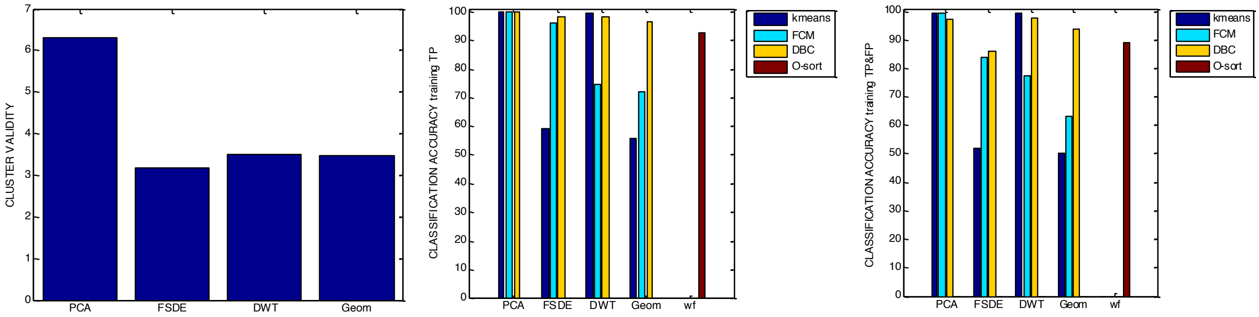

Figure 4: Figures of the results of the automatic comparison of all the combinations of algorithms

‘REAL data’: Off-line Spike sorting GUI for real multichannel signal analysis off-line.

Figure 5 depicts the layout of the GUI for the offline analysis on real single or 60-channels MEA signals.

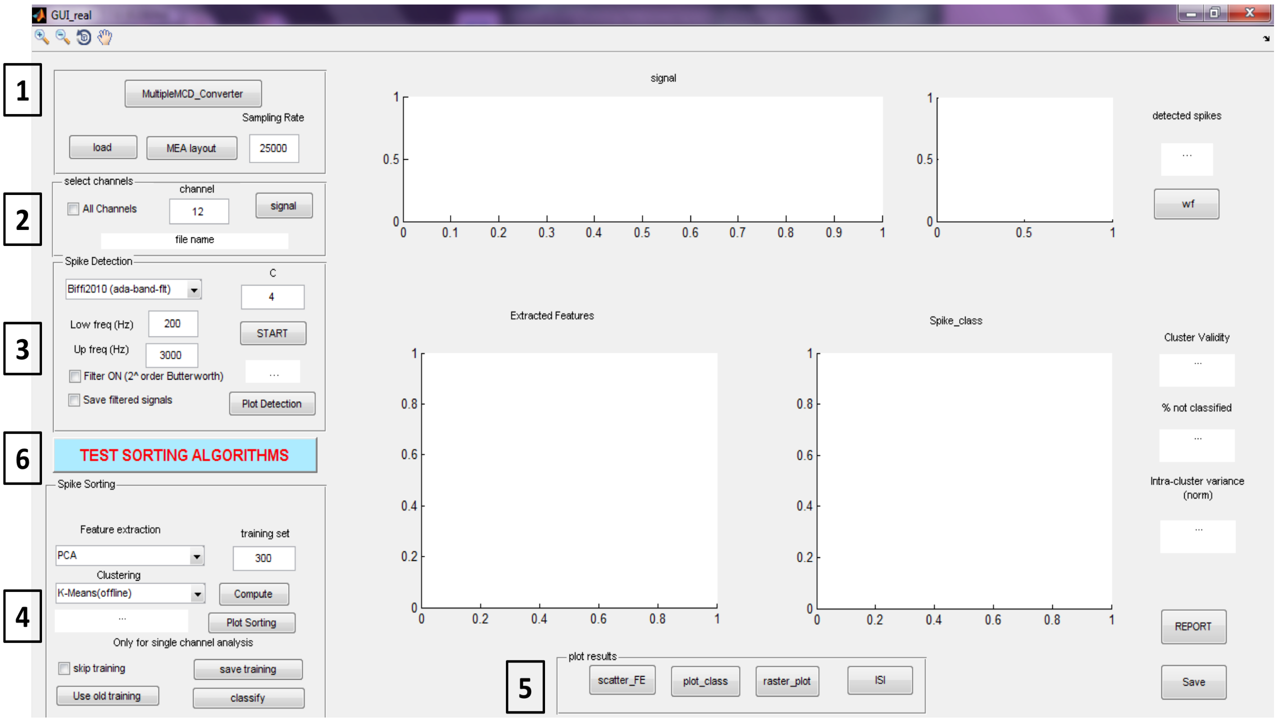

Figure 5: GUI to import and analyze real MEA signals

MultipleMCD_converter in **[1]** performs the conversion from MCD file format to 60 .mat single signals taking advantage of the Neuroshare library. Each signal is saved in a new directory named after the .mcd file analyzed, automatically created.

At the end of the conversion the button turns green.

As in the test data interface, the user has to load the data (the 60 signals). The loaded signals can be plotted on the classic MEA layout (Fig. 6).

In **[2]** it is possible to choose the single channel to analyze *(and the GUI works like the Test Data GUI one, except for the results visualization)* or by selecting the option ‘**all Channels**’. A GUI is presented (Fig 7), which allows the user to select the channels for a simultaneous analysis (**ALL** selects all the 60 signals).

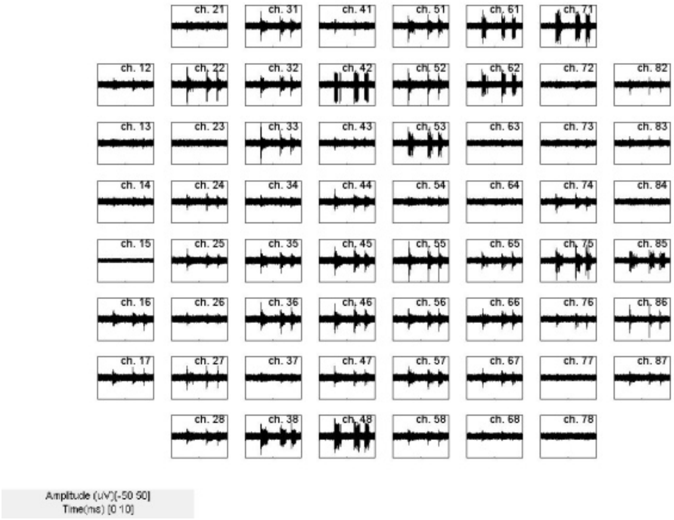

Figure 6: the 60 MEA signals plotted in a MCR layout style

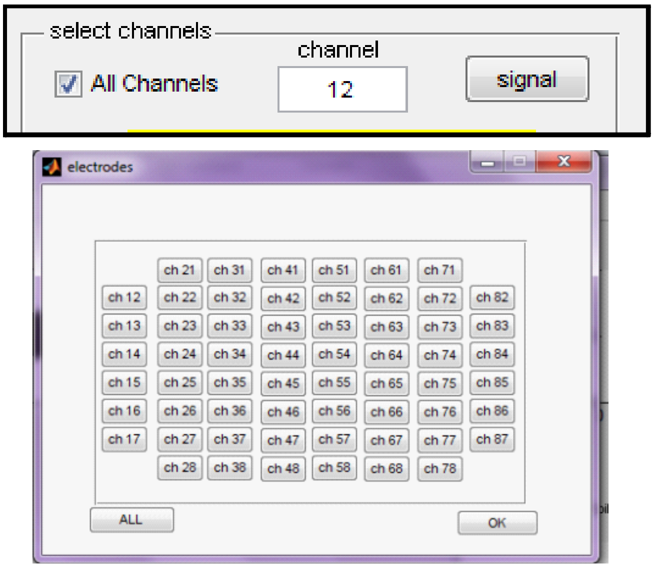

Figure 7: electrodes selection GUI

Anyway it is possible to visualize the sorting results on single channel even when the spike sorting is carried out on several channels at the same time .

[3] is the spike detection panel. If it is necessary the data can be filtered and saved by checking the relative selection check boxes (a folder for the filtered signal is created). The frequency band is settable by the user.

If the **All Channel** label is on, the detection results are automatically saved in a newly created directory as .mat files named 'filename_nchannel_detection' (containing t_stamps, index_stamps, template and channel firing rate and the extracted spikes).

Plot detection opens the dialog GUI in Fig. 8 to select what to plot: **filtered signals** in the MEA layout or the **rasterplot**. **Spikes** opens other options: the user can plot the **waveforms** per channel, the **number of spikes** (displayed also as a colored map superimposed to the MEA layout) or the **firing rate** per channel. All the plot are automatically saved as .jpeg figure in a directory selected by the user (*which has to be different from the ones in which the detection results are automatically saved*).

[4] groups the **spike sorting** parameters and methods selection. The chosen algorithm is applied to all the selected channels ('**compute**') and the results are automatically saved. For each channel the sorting result is saved as a .mat file named 'filename_nchannel_sorting' containing : ID, FE, axis, CV value, mean intracluster variance (ICV) and the sorting parameters.

As for the simulated data, the training can be saved, but only for single channel analysis and the automatic selection of feature can be matched only with K-means.

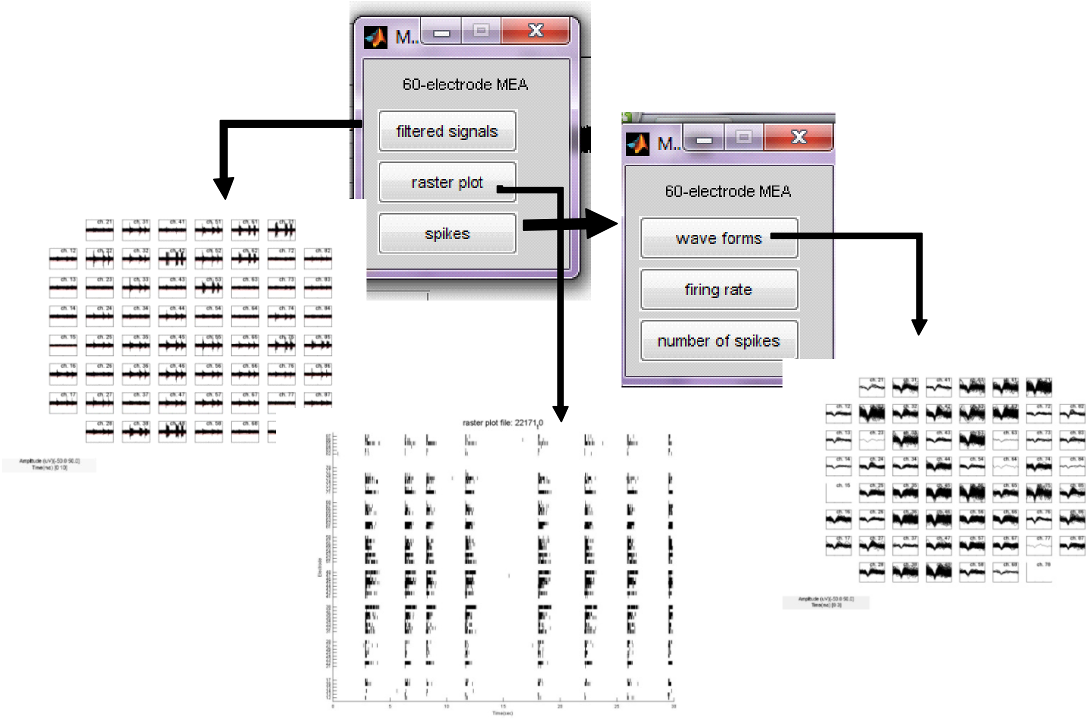

Figure 8: GUIs for plot options

NOTE: after the spike detection it is possible to generate a report (.txt) in which there are information about the detection (parameter chosen) and the location of the results (directory path).

Using the buttons in **[5]** the user can visualize the sorting outcomes for every channel, like in figure 9 (top). Otherwise the command '**plot sorting**' in **[4]** allows to plot the analysis results in the classic MEA layout or the sorted raster plot (Fig. 9, bottom)

The **ISI** button calculates the inter-spike interval for each channel and saves the results (.mat files) for each channel.

A **report** can be generate reporting the main information about the completed analysis.

Finally, the 'TEST SORTING ALGORITHMS' **[6]** provides an automatic comparison of all the possible combination of FE and clustering methods on a SINGLE CHANNEL.

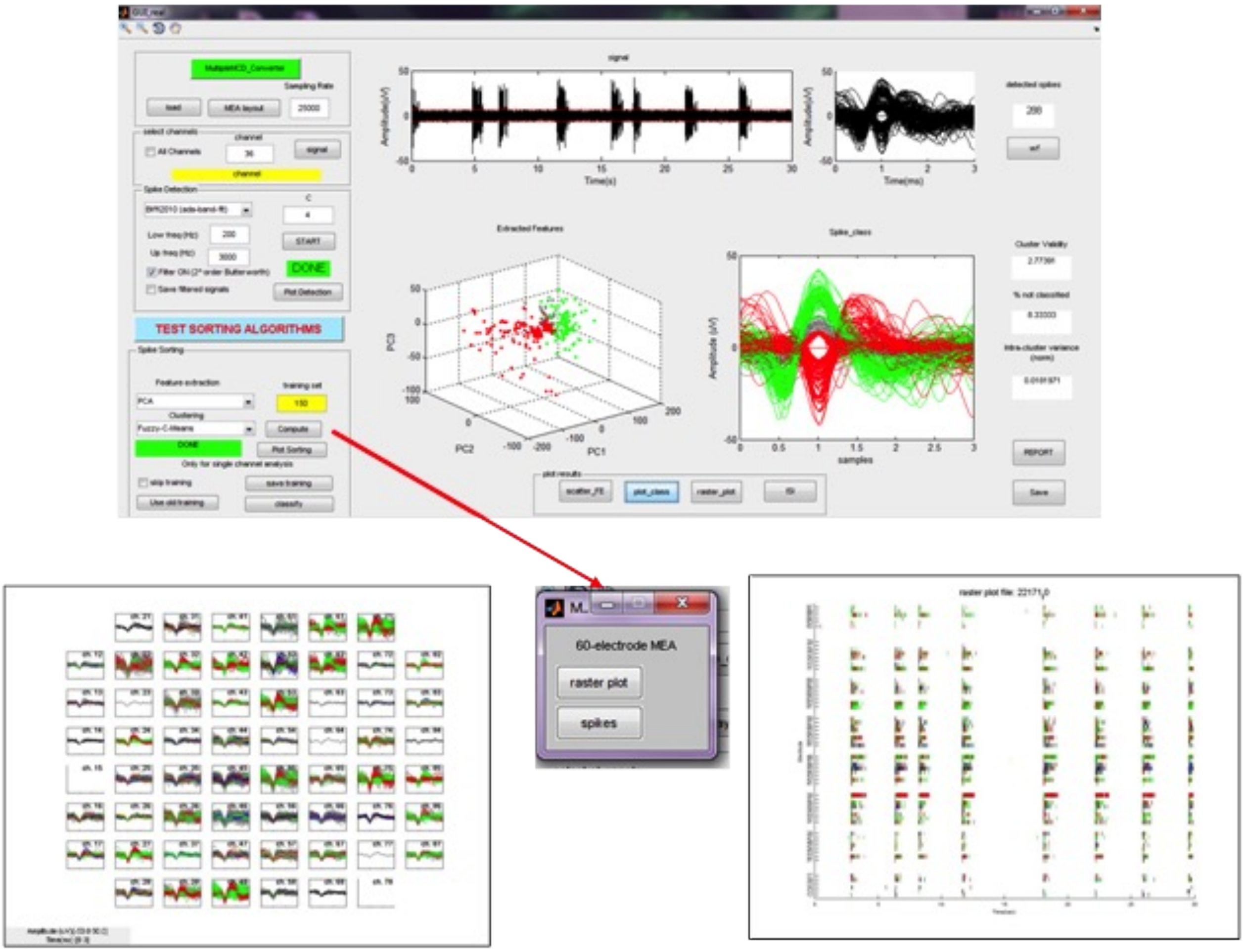

Figure 9: example of sorting results; for a single channel (top) and for all the electrodes (bottom)

'REAL data': on-line Spike sorting GUI for real multichannel signal analysis on-line.

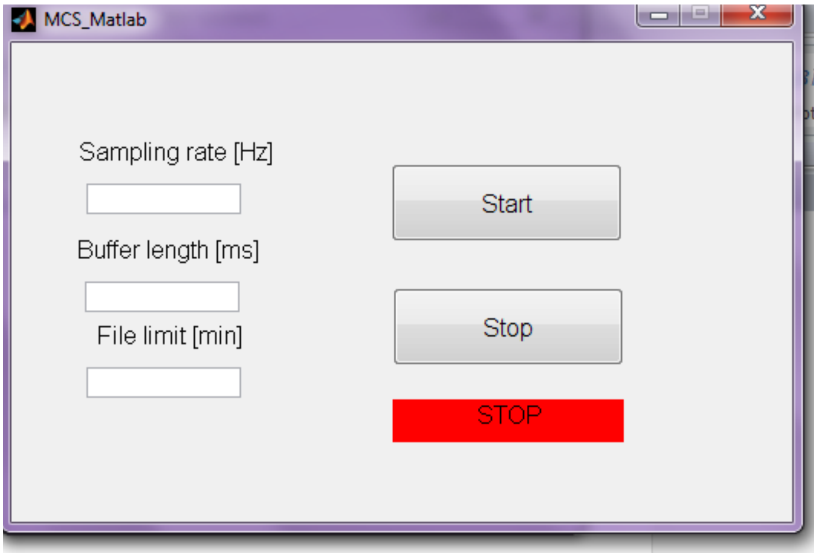

Figure 10: GUI for on-line analysis directly in Matlab

NOTE! This part of the framework currently works only in conjunction with two kinds of 60 channel A/D device from Multi Channel Systems GmbH (i.e., USBME64 and USBME2100).

The GUI in Fig.10 allows to set the sampling rate, the transmission buffer length and the maximum duration (min) of a recording to be saved in a single file. Then, the START button launches a script which establishes the real-time communication with the MEA device and perform spike detection, spike sorting and storage of results. The current version is not completely automatic, in order to let an expert user to easily modify the script code to test runtimes, select which method to apply, load training data, optimize the code, etc.

Note:

Data are saved as binary files which can then be read by the "read_bin_files.m" script.
